# ESR paper on structured reporting in radiology

**DOI:** 10.1007/s13244-017-0588-8

**Published:** 2018-02-19

**Authors:** European Society of Radiology (ESR)

**Affiliations:** European Society of Radiology (ESR), Neutorgasse 9/2, 1010 Vienna, Austria

**Keywords:** Radiology trends Q000639, Radiology standards Q000592, Radiology statistics & numerical data Q000706, Automation D001331, Communication D003142

## Abstract

**Abstract:**

Structured reporting is emerging as a key element of optimising radiology’s contribution to patient outcomes and ensuring the value of radiologists’ work. It is being developed and supported by many national and international radiology societies, based on the recognised need to use uniform language and structure to accurately describe radiology findings. Standardisation of report structures ensures that all relevant areas are addressed. Standardisation of terminology prevents ambiguity in reports and facilitates comparability of reports. The use of key data elements and quantified parameters in structured reports (“radiomics”) permits automatic functions (e.g. TNM staging), potential integration with other clinical parameters (e.g. laboratory results), data sharing (e.g. registries, biobanks) and data mining for research, teaching and other purposes. This article outlines the requirements for a successful structured reporting strategy (definition of content and structure, standard terminologies, tools and protocols). A potential implementation strategy is outlined. Moving from conventional prose reports to structured reporting is endorsed as a positive development, and must be an international effort, with international design and adoption of structured reporting templates that can be translated and adapted in local environments as needed. Industry involvement is key to success, based on international data standards and guidelines.

**Key Points:**

• *Standardisation of radiology report structure ensures completeness and comparability of reports.*

• *Use of standardised language in reports minimises ambiguity.*

• *Structured reporting facilitates automatic functions, integration with other clinical parameters and data sharing.*

• *International and inter-society cooperation is key to developing successful structured report templates.*

• *Integration with industry providers of radiology-reporting software is also crucial.*

## Introduction

In 2006, Michael Porter published his book “Redefining health care” [1] and in 2009 and 2010 he published additional articles regarding creating value in health care [2, 3]. In these publications, the author described the need for change in health care systems from volume-based to value-based care. Similarly, the American College of Radiology introduced Imaging 3.0™ as a strategic initiative to introduce the concept of the imaging value chain. Boland et al. published about this topic in the Journal of the American College of Radiology [4, 5] and pose the question of how radiologists can add value to patient care. One key way radiologists can ensure the value of our contribution is to optimise the impact of our reports [6].

The ESR has recently published a concept paper on Value-Based Radiology; one of the proposed key factors relates to addressing report characteristics, including structured reporting [7]. The American Recovery and Reinvestment Act and the Health Information Technology for Economic and Clinical Health stated that structuring data in health records will lead to an important improvement in patient outcomes [8]. As radiology reports are part of the health record, the current format of free-text radiology reports should be “organised” and shifted toward structured reports. Still, the question as to whether all radiological examinations should have a structured report and if so the actual report structure remains open.

In 2007–2008, RSNA started a Structured Reporting initiative as one of the pillars of the RadLex steering committee actions. The RadLex project (“lexicon for uniform indexing and retrieval of radiology information resources”) is a collaboration involving RSNA and other radiology organisations, including the ACR (American College of Radiology), subspecialty societies and numerous physicians, aimed at developing a comprehensive radiology lexicon.

The need to use uniform language and structure to accurately discuss findings in radiology was the basis for developing the concept of “structured reporting”.

This article provides an overview of the definition of structured reporting, the reasons why it is valuable, and the requirements to implement it in radiology.

## Definition

Weiss et al. describe three levels of structured reporting [9]:

1. The first level is a structured format with paragraphs and subheadings. Currently, almost all radiology reports contain this structure, with sections for clinical information, the examination protocol, radiological findings and a conclusion to highlight the most important findings.
2. The second level refers to a consistent organisation. For example, a knee MRI describes all relevant anatomic regions such as cruciate ligaments, menisci, collateral ligaments, and so on, with an internal logical order.
3. The third level directly addresses the consistent use of dedicated terminology, namely standard language. To increase the accessibility and reusability of radiology reports, defined terms of a standardised lexicon should be used.

## Reasons for structured reporting

The main functional needs for moving from traditional free text reporting to standardised and structured reporting can be addressed under three categories: quality, datafication/quantification and accessibility.

### Quality

A critical quality improvement resulting from the use of structured reports is standardisation. The use of templates in structured reporting provides a checklist as to whether all relevant items for a particular examination are addressed. This will ensure that answers are provided to the clinical question that motivated the procedure. Some of these questions will be quite general, i.e. the same for any instance of a particular procedure (e.g. key measurements of liver in ultrasound examinations), whereas some of them can be very case dependent. Prompt, clear communication of the information to the ordering clinician should always be kept in mind. Furthermore, the use of standardised terminology prevents ambiguity and facilitates comparability of disease states and treatments. This will provide greater guidance to the referring physicians to deliver the appropriate care. Additionally, use of standardised reporting of techniques facilitates ease of comparison of results (for research and clinical practice).

### Datafication/quantification

Depending on the reporting radiologist’s practice, free text reports may not clearly respond to the modern clinical question. Radiology should provide patient-tailored reports containing key data elements and relevant quantified parameters, i.e. imaging biomarkers or the so-called “radiomics” [10]. Through this “structure” the report will allow the integration of tools that help radiologists in reporting with relevant information (e.g. automatic TNM classification) and recommendations based on current available literature. A potential future extension of the report could be the integration and combination of radiology data elements with other key clinical parameters (e.g. laboratory results), leading to an integrated and precise diagnosis, and beyond, to computer-assisted clinical decisions. Such decisions can concern all aspects of patient management in personalised medicine, e.g. diagnosis, selection of optimal treatment (including prediction of risks) and evaluation of treatment response [11]. They can also help in assessing if a patient is eligible for some clinical trial by automatically assessing if the inclusion criteria are met. Furthermore, data could be easily shared with data registries, e.g. national cancer registries, biobanks, and so on. It is also possible that, as in a Google-like search and data analysis, the structured reports will be used to develop artificial intelligence algorithms by annotating cases that will ultimately facilitate/support the tasks of the radiologist. All these advanced capabilities require that the report be captured and linked to well-defined vocabulary such as RadLex.

### Accessibility

Radiology reports contain information that can feed multiple processes: diagnosis, of course, and, more generally, many clinical decision processes, as explained above. Besides, radiology reports constitute a precious information source for the evaluation of the quality and effectiveness of radiology (including the value radiology adds to patient care) through the derivation of important metrics [12]. Finally, radiology reports can be seen as a rich source of information for research, e.g. research on imaging biomarkers, because they allow linking of qualitative and quantitative information about patient pathophysiology to descriptions of clinical states and symptoms. This allows automated data mining, which may help to validate the relevance of imaging biomarkers by highlighting the clinical contexts in which they are most appropriate and helping to devise potential new application domains. For this broad use of information contained in reports to become a reality, radiology reports must be structured with a content based on a standard terminology, and they must be accessible via standard access mechanisms and protocols. Moreover, the data must be stored in a suitable format that makes it easily accessible for any authorised party who needs to extract any appropriate or desired information. Examples of such situations include extraction of some significant observations from a computer-assisted detection (CAD) report to include them in the final report, assessing the relevance of a particular case with respect to clinical trials performed locally, clinical research, inclusion into pathology-specific imaging biobanks and big data learning [3]. Indeed, current implementations of reporting software often keep the structured content of the reports in proprietary databases and deliver and share only non-structured documents (e.g. pdf format), thus jeopardising any kinds of reuse by other applications. Such situations may be found, e.g. with specialised applications dedicated to the assessment of patient treatment response (such as RECIST based) or to speech management and recognition. Actually, all applications involved in either the creation or utilisation of structured reports should constitute a robust ecosystem, supporting both sophisticated radiology reporting and decision support systems, as demonstrated in [13].

## Requirements

To fulfill the needs listed in the previous section, the following three aspects with the according recommendations need to be provided: (1) definition of the actual content and structure of the structured reports; (2) choice of the standard terminologies to encode the information; (3) tools and protocols that should be used to create the reports (potential use of voice recognition versus other modes of data entry) and to exchange report templates and structured reports (DICOM, HL7, IHE MRRT).

### Definition of content and structure of reports

This aspect is probably the major difficulty of the undertaking, for many reasons:

1. the structured reports must match the clinical requests, i.e. include what is appropriate in the specific context, not more not less;
2. the structured report must be responsive to the specific clinical circumstances; for example, a report describing a rectal cancer must include all surgically relevant information, such as for instance mesorectal involvement;
3. medical procedures and clinical situations must be categorised, which introduces a strong dependence on the codification aspect (addressed in next section);
4. it is necessary to define and agree on which authority is legitimate to define such structured reports: it can be a standardisation organisation (the DICOM Standards Committee actually defined many specifications of structured reporting, which is legitimate since DICOM has contributors from many medical professional societies) but structured reports may also be defined directly by medical professional societies, national and international;
5. there may also be additional difficulties related to intellectual property;
6. all this is in constant evolution, especially the introduction in structured reports of imaging biomarkers, which is highly dependent on imaging technology and on the availability of consensual clinical decision models that use them.

The use of standardised reporting criteria includes the following:

- Staging systems (TNM, etc.) [N.B., this may include several potentially valid staging schemes that are used based upon regional practice; thus, it is imperative to specify which system forms the basis of the report];
- Procedural components (i.e. adhering to consensus documents such as the Standards of Reporting Criteria for Tumor Ablation [14].
- Complications (for procedures)
- Possibility to include quantitative biomarker information. In this complex context, RSNA has been working on a template report library since 2008 [15]. The structured reports are stored at the website radreport.org,^1^ which contains templates developed with the collaboration of subspecialty societies and coded with RadLex terms. The RadLex vocabulary and codes allow extraction of terms and also linkage between different languages. This initiative is now common between RSNA and ESR, with a joint committee (the “Template Library Advisory Panel, TLAP) that acts as an editorial board and reviews the templates’ content and structure. The open.radreport.org platform offers the possibility to all ESR and RSNA members to propose their own structured reports and to rate and comment on templates proposed by others.

The reports that fulfill the quality requirements will be included to the general radreport.org template library. In addition to the above-mentioned criteria, the structured reports should be succinct, with content specifically directed to the context and purpose of the examination. Text grouping within the report should be logical with a limited number of fields and internal logic to minimise the number of fields. The field structure should always allow integration of the same type of information under the same fields to allow its easy retrieval.

When developing structured reports, it should be noted that there is a distinction between “general reports” that apply to any instance of a given type of imaging procedure and specific reports, which may also depend on each individual case and on the specific questions the referring physician (or clinical research study) might have.

### Standard terminologies

As soon as structured reporting is considered beyond the second level (consistent organisation) defined earlier (Definition section) codification using some controlled terminology is involved. The difficulty here arises from the very broad domain that must be covered, not limited to imaging procedures, techniques and semiology, but encompassing also clinical context, definition of diseases, syndromes, symptoms, and so on [13]. Moreover, there is also a legitimacy issue in terms of knowing which institution is best positioned to define some controlled terminology. In practice, these terminologies (e.g. SNOMED CT, LOINC, FMA, and so on) were defined by multiple organisations (IHTSDO, Regenstrief Institute with Indiana University, Washington University in Seattle, respectively), with significant overlaps in terms of scope. To remedy this situation, efforts such as the Unified Medical Language System (UMLS) project, initiated by the National Library of Medicine in the US, managed to introduce mappings and concept unique identifiers (CUI), which allow relating common concepts across multiple terminologies [16]. Besides, the DICOM Standards Committee made a choice for SNOMED as its primary source of terminology and created its own terminology resource (named DICOM Controlled Terminology) when the needed terms cannot be found from existing resources. In parallel, new methods, tools and standards emerged from the cross-fertilisation of activities of the knowledge representation community with the world wide web, leading to the development of ontologies and other semantic web technologies. The radiological community understood very early the new possibilities that may arise with this technology, especially in terms of automated processing and reasoning. RSNA launched the RadLex project, which aimed at gathering a complete lexicon for radiology, made of terms borrowed from multiple terminological sources, but integrated into a common resource [17]. RadLex contains terms from multiple ontologies and is itself implemented in OWL (the Web Ontology Language) [18], but still suffers from several drawbacks that limit its possibilities in terms of reasoning and make maintenance somewhat cumbersome. This brief historical reminder highlights the main difficulties of designing terminological resources for radiology in general and structured reporting in particular. One of them is the difficulty in specifying the required level of expressivity, so that reliable reasoning can be implemented, which is needed at the minimum to be able to check the consistency of the terminology, and beyond, to ensure the reliable implementation of clinical decision models.

### Implementation with suitable tools and protocols

Three major aspects have to be addressed: (1) reporting tools and their integration in the radiology workflow; (2) data formats used to represent radiology reports; (3) protocols to communicate structured reports.

- Reporting tools and their integration in the radiology workflow

To get structured reporting working in daily practice, it should be incorporated in the current workflow as much as possible and needs to be able to work well in practice. This means that structured reporting solutions should ideally be incorporated in a voice-driven workflow, as many radiologists use microphone technology to create their reports. Furthermore, templates should be linked to imaging codes so that the correct template appears when starting a new dictation [13]. In practice the design of a structured reporting tool is very challenging in terms of usability [19], and for many more reasons: the need to avoid human errors, the need for automatic management of dependent components of the report and the need for flexibility in importing precise image references and observations from companion reports (e.g. measurements, observations from CAD reports), and so on. Moreover, such capabilities often require interoperability with other information systems such as Computerised Physician Order Entry systems (to access details of the clinical request), or Radiology Information Systems (e.g. to automatically retrieve the relevant reporting template, as well as the description of the image acquisition and image processing processes) or with the Electronic Patient Record. In fact, the products available on the market have not yet reached a sufficient level of usability, thus discouraging most radiologists from using them. More efforts are needed on the manufacturers’ side to provide more mature products.

(b) Data formats used to represent reporting templates and radiology reports.

The Integrating the Healthcare Enterprise (IHE) initiative defined an integration profile dedicated to the provision of reporting templates called Management of Radiology Report Templates (MRRT). In MRRT reporting templates are provided in HTML5, a syntax that enables direct management by web browsers. The data structure is flexible. It allows both management and free text and coded entries, e.g. using RadLex. Basic metadata use terms from the Dublin Core metadata element set, a standard terminology for document metadata [20]. This choice strikes a balance between expression of coded content and description of a user interface and the related data capture methods [21, 22].

Another complementary approach relies on Common Data Elements (CDE). These are “elements that are collected and stored uniformly across institutions and studies and are defined in a data dictionary” [23]. Basically, a CDE specifies the name of the entity and its data type, lists the allowable values and associates a question that may be prompted to the user to solicit selection of a value. CDEs have been defined for various domains of medicine such as cancer, stroke and epilepsy. Recently, a proposal was made to use CDEs to support authoring of report templates such as those published by the RSNA [24]. Concerning radiology reports, several standard formats currently exist. The DICOM standard introduced structured reporting in 1999, with DICOM Supplement 23 Structured reporting object. This supplement introduced several generic models of reports of various complexity, namely Basic Text SR, Enhanced Text SR and Comprehensive SR, the latter two allowing the inclusion of coded observations and measurements. This extension of DICOM did not lead to a wide implementation by industry, but it paved the way for the definition of more specific structured reports, e.g. dedicated to CAD reports (mammography CAD, chest CAD, colon CAD) as well as various procedure reports (e.g. catheterization laboratory SR, vascular ultrasound procedure report, echocardiography procedure report). Health Level Seven (HL7) standards also provide specifications for reporting templates and radiology reports, based on the Clinical Document Architecture (CDA). Especially DICOM Part 20 (Imaging reports using HL7 Clinical Document Architecture) specifies templates for the encoding of imaging reports using CDA as well as a transformation to translate DICOM structured reports into CDA documents.

(c) Protocols to communicate reporting templates and reports.

The IHE MRRT integration profile specifies the roles of the different actors involved in creation of imaging report templates, namely Report Template Creator, Report Template Manager and Report Creator, bearing the roles of creating the templates, of managing them and using them to create actual imaging reports, respectively. Communications between these actors rely on the HTTP protocol. As for DICOM structured reports, they can be exchanged with both traditional DICOM messaging (Storage and Query/Retrieve) and the DICOMweb set of RESTful services over HTTP, more adapted to the access to images and reports from web browsers.

Several reporting tools were developed based on IHE MRRT. For example, Karos Health developed T-Rex as open-source freeware for RSNA and the user community. Similarly, the University of Mainz developed a web-based, open-source reporting platform with a generic interface that allows the import of any MRRT-compliant HTML5 template found on the RSNA’s radreport.org website [25].

## Implementation strategy

An important requirement for successful implementation of structured reporting is to respect current radiologists’ workflow. This means that structured reporting functionality should include speech-driven reports. Furthermore, to gain support among radiologists, it is essential that radiologists understand the importance and added value of structured reporting. When defining a template for a specific disease, it is crucial that all relevant radiologists are involved and that referring clinicians can give their opinion. To gain experience with developing a template and applying structured reporting in clinical practice, a staged approach including a pilot introduction among enthusiastic first adopters can accelerate the broader introduction of structured reporting. Furthermore, it can be most beneficial to involve trainees and residents in the implementation strategy. Also, for successful implementation it is important to provide the flexibility that will enable one to easily change the templates, so that the end users (i.e. residents and radiologists) are as comfortable as possible when using structured reporting. Finally, the usage of structured reporting should be encouraged by national societies and subspecialty societies and ideally could be part of quality audits for radiology departments.

## Summary and roadmap

Wide adoption of structured reporting is of critical importance for providing referring physicians and ultimately patients with the best quality of service, for assessment of radiology’s contribution and fair recognition of radiology in value-based medicine and for providing researchers with the best quality data in the context of big data exploitation of available clinical data.

Implementation is complex for all the reasons detailed above. It has a major impact on radiologists’ daily practice, and it requires mature technology to successfully address the pending user-friendliness, organisational and interoperability challenges, especially adequate storage of data, and easy and adequate connections with PACS- and post-processing software. Consequently, introduction of structured reporting should be seen as a comprehensive effort, affecting all domains of radiology.

Therefore, all radiologists and radiology departments should endeavour to progressively move from "prose" reports to structured reports, in adherence with consensus documents based on international standards, and this effort should be supported by active education and training programmes (e.g. through dedicated sessions at the European Congress of Radiology (ECR), training curricula for young radiologists). Especially subspeciality societies and national societies are encouraged to design and adopt SR templates. As an incentive, health authorities and reimbursement parties may condition reimbursement of imaging medical acts to the delivery of SR reports.

Of course, collaboration at the international level in needed on the design and adoption of common SR templates (e.g. TLAP) and should be promoted and facilitated.

As for the necessary adoption of common reporting templates, the current RSNA and the joint ESR-RSNA efforts are a first step. A next step should try to rationalise the organisation of SR templates through a logical organisation based on the ontology-based classification of clinical situations and imaging procedures. The adoption of multilingual templates should be systematically preferred to avoid the quantitative explosion of the number of templates. These technical aspects are complex and should be addressed in collaboration with organisations developing standard terminologies/ontologies. In particular, reporting templates should increasingly include quantitative measurements (imaging biomarkers), especially when formalised clinical decision models exist, which are based on these measurements.

Finally, the wide scale adoption of structured reporting cannot be envisaged unless standard-based and user-friendly reporting tools are provided by industry. It is also the radiologists’ responsibility (as customers) to choose those products that follow international standards (DICOM, HL7) and implementation guidelines (IHE) for the exchange of SR templates and SR reports so that a significant evolution of the market can take place.

## Footnotes

1 radreport.org metrics can be viewed at: http://radreport.org/metrics.php
